# COVID‐19 vaccine: recent advancements and future prospects

**DOI:** 10.1002/mco2.646

**Published:** 2024-07-05

**Authors:** Li Xu, Min Li, Wu He

**Affiliations:** ^1^ Center for Drug Evaluation National Medical Products Administration Beijing China

## Abstract

Nowadays, although severe acute respiratory syndrome coronavirus 2 (SARS‐CoV‐2) epidemic has been handled with long‐term management globally, the virus strains keep continuous evolution in an uncertain direction. The newly emerged JN.1 dominated the globally circulating variants in a short time and resulted in increasing hospitalizations. Up to 2024, variant vaccines with the composition of XBB sub‐lineage were available due to the coordinated efforts of developers and regulatory agencies. The development of updated vaccines is still needed and the regular availability of coronavirus disease 2019 (COVID‐19) vaccines should be timely guaranteed. The current landscape of COVID‐19 vaccines and the strategies for accelerating vaccine development and approval are reviewed. Proposals to enhance variants monitoring and the establishment of the strain recommendation mechanism are made. This review provides suggestions about platform technology designation and application, real‐world data leveraging and modification to regulatory pathways both for the post‐pandemic era of SARS‐CoV‐2 and for the future unknown pathogen pandemic.

## 1 INTRODUCTION

On May 5, 2023, the World Health Organization announced that coronavirus disease 2019 (COVID‐19) is no longer considered a public emergency of international concern health events.^1^ Subsequently, several countries decided to move from emergency response to long‐term management.

SARS‐CoV‐2 continues to mutate, from the early Alpha, Beta, Gamma, Delta, Omicron BA, and XBB to the recent JN.1. The currently dominant circulating variant JN.1^2^ is derived from BA.2.86, which has a large number of mutations. BA.2.86 is evolutionary and antigenically distinct from the previously dominant variants of XBB sub‐lineages. So far, the rules of mutation and prevalence of SARS‐CoV‐2 are not yet fully understood. Due to the rapid genetic and antigenic evolution of SARS‐CoV‐2 variants, cross‐neutralization following vaccination and/or infection and the effectiveness of approved vaccines may decrease in the face of circulating SARS‐CoV‐2 variants. Some studies believe that the reinfection rate of the SARS‐CoV‐2 will reach more than 65%, and those who are reinfected will increase the risk of all‐cause death and hospitalization and will have sequelae.^3^, ^4^ Furthermore, the number of infections increases the risk of cumulative sequelae. There is a need for ongoing research and development of effective COVID‐19 vaccines.

This paper retrospect the strategies for accelerating development and approval, guaranteeing the availability of COVID‐19 vaccines to respond the public emergency and the transition phase to normal management, and propose the follow‐up measures to be taken for both SARS‐CoV‐2 variants epidemic and future unknown pathogens pandemic.

## 2 CURRENT LANDSCAPE OF COVID‐19 VACCINES

Depending on years of continuous iterative development, COVID‐19 variant vaccines with the XBB strain composition have been authorized/approved by relevant national regulatory agencies. Challenges and limitations from the technology, relevant infrastructures, SARS‐CoV‐2 variant and regulatory decision‐making strategies affect the development and research from the initial protype to the variant vaccines. Lessons about national policy and mechanism, laws, procedures, technical guidelines, and platform technology are obtained.

### 2.1 Summary of COVID‐19 vaccines newly authorized/approved

Commercial vaccine manufacturers and other entities use different technologies to develop COVID‐19 vaccine candidates. After the authorization of the prototype vaccines and series of follow‐up updating variant vaccines, the currently mainly authorized/approved available COVID‐19 variant vaccines include recombinant protein subunit vaccines, adenovirus vector vaccines, and mRNA vaccines (Table 1).

**TABLE 1:** Summary of approved/authorized coronavirus disease 2019 (COVID‐19) vaccines containing XBB lineages.

			Regulatory agency			
Platforms	Vaccines	Manufacturers	FDA	EMA	MHRA	NHC and NMPA
Recombinant protein subunit (adjuvanted)	Novavax COVID‐19 vaccine, adjuvanted (2023‒2024 formula)	Novavax, Inc.	EUA: 03/10/2023	(Adapted) authorization: 31/10/2023	‒	‒
	SCV01E‐2	Sinocelltech, Ltd.	‒	‒	‒	EUA: 01/12/2023
	Recombinant COVID‐19 trivalent (XBB + BA.5 + Delta) protein vaccine (Sf9 cell)	WestVac Biopharma	‒	‒	‒	EUA: 08/06/2023
	Recombinant COVID‐19 (XBB) trimer protein vaccine (Sf9 cell)	WestVac Biopharma	‒	‒	‒	EUA: 01/12/2023
	Recombinant COVID‐19 bivalent original/Omicron XBB (CHO cell)	Livzon Mabpharm Inc.	‒	‒	‒	EUA: 01/12/2023
mRNA	Moderna COVID‐19 vaccine (2023‒2024 formula)	Moderna TX Inc.	EUA: 11/09/2023 BLA (supplement): 11/09/2023	(Adapted) authorization:31/08/2023	Marketing authorization: 15/09/2023	‒
	Pfizer‐BioNTech COVID‐19 vaccine (2023‒2024 formula)	Pfizer, Inc.	EUA: 11/09/2023 BLA (supplement): 11/09/2023	(Adapted) authorization: 15/09/2023	Marketing authorization: 15/09/2023	‒
	Bivalent (XBB.1.5 + BQ.1)	CSPC Pharmaceutical Group Limited	‒	‒	‒	EUA: 01/12/2023
	RQ3033	WALVAX	‒	‒	‒	EUA: 01/12/2023
Adenovector	Convidecia Air XBB.1.5	CanSino Biological Inc.	‒	‒	‒	EUA: 01/12/2023

Abbreviations: FDA, Food and Drug Administration; EMA, European Medicines Agency; EUA, Emergency Use Authorization; MHRA, Medicines and Healthcare Products Regulatory Agency; NHC, National Health Commission of the People's Republic of China; NMPA, National Medical Products Administration.

The mainly updating variant vaccines authorized by Food and Drug Administration (FDA) were based on Moderna and Pfizer‐BioNTech's mRNA vaccines and Novavax's protein vaccine.^5^, ^6^, ^7^ For the XBB.1.5 formulation, both the above two mRNA vaccines were authorized emergency use authorization (EUA) and approved with a supplement to the BLA for different ages. In early phase, European Medicines Agency (EMA) authorized one inactivated, two vector‐based, two mRNA, and three adjuvanted recombinant protein vaccines, updating approval of variant vaccines were based on Moderna and Pfizer‐BioNTech's mRNA. TheMedcines and Healthcare Products Regulatory Agency (MHRA) and Pharmaceuticals and Medical Devices Agency (PMDA) are basically in sync with the FDA and EMA on the iterative routes and rhythms of variant vaccines. In addition, PMDA approved two early‐strain mRNA vaccines in the second half of 2023, which seemed to be approvals of technical platforms reserves for the future.^8^

In 2023, the year after the adjusting of national strategy for prevent and control of SARS‐CoV‐2 in China, multiple COVID‐19 variant vaccines were authorized for emergency use. These variant vaccines covered series of variants and formulation, including multivalent and monovalent, mRNA, adenovirus vectored and adjuvanted recombinant protein vaccines, composition of Alpha, Beta, Delta, Omicron BA.1, BA.4/5, BQ.1.1, XBB.1, and XBB.1.5.

### 2.2 Challenges and limitations in vaccine development and approval

Strategies for the development of vaccines, including selection of strains, selection of technical routes and antigen design are all determined by the developers independently. Especially for strains selection, no unified strain recommendation mechanism has been established yet, unlike influenza. Due to the heterogeneously variant circulation across different regions, different human immune background, currently uncertain rules of strain mutations, various technical routes, available vaccine safety, and effectiveness data, it will still be a challenge to unify the updating of variant vaccines globally.

In the early phase, developing vaccines depending on existed technique platforms was the fastest way, but the existed platforms might be not suitable for the COVID‐19 vaccines. For a newly built technology platform, it takes time from establishment to maturity, which might be a promising platform for COVID‐19 vaccines. It is difficult to transform laboratory basic research projects into industrial, which need a solid basic research and development basis with years of technology accumulation and rapid industrially transforming supporting system. Meanwhile, the manufacturing facilities required are usually not ready‐made, and it will take time to build new infrastructures. The choice of industrial scale is also a challenge, which needs to balance technical capabilities, hardware facilities, vaccine supply and demand, and the competition for time. Due to the mutation of virus, the current circulating strain is usually no longer relevant to the original composition of the product when clinical trial data obtained, which makes difficulties in regulatory decision making according to the clinical use‐oriented approach.

The rapid, irregular, and constantly mutation with immune escape of SARS‐CoV‐2, leading to narrow the time window of preclinical development, review and approval, and clinical trials and manufacturing. On the one hand, it is difficult for developers to formulate development strategies, and the formulation of Target Product Profile (TPP) is quite a challenge. It may be necessary to keep developing variant vaccines continuously, invest a lot of manpower and material resources to catch the time and variant. On the other hand, all aspects of review and approval need to be accelerated, which need the support by corresponding processes and regulations. Acceleration of approval could not reduce the approval process or lower the standards to guarantee the quality of vaccines.

### 2.3 Lessons from pandemic on vaccine development and approval

It is crucial to lay out different technical routes for vaccine development nationally at the beginning of the pandemic, which promotes the development and establishment of innovative technology platforms and built a vaccine development echelon to achieve sustainable iteration and clinical trial. For instance, China initially displayed technical routes including inactivated, mRNA, virus vectored, and adjuvanted recombinant protein vaccines. Three inactivated, one adenovirus vectored and one recombinant protein subunit original COVID‐19 vaccines were granted conditional marketing authorization in China. Recently, authorized variant vaccines include adenovirus vectored, adjuvanted recombinant protein, and mRNA vaccines. As of the adenovirus vectored routes, both injected and aerosolized inhalation variant vaccines were developed. Recombinant protein vaccines major the authorized COVID‐19 vaccines, including CHO cell and Sf9 cell expression systems, dimeric and trimeric proteins, monovalent and multivalent, aluminum adjuvants, and oil‐in‐water emulsion adjuvants.

The establishment of a joint prevention and control mechanism coordinates and ensures efficient cooperation among all relevant parties, integrates resources, ensures distribution and supply, and shortens the overall time from vaccine development to availability. At the beginning of 2020, the Chinese government established the Joint Prevention and Control Mechanism of the State Council, a multi‐ministerial coordination working mechanism platform, and established a dedicated vaccine research and development working group of the Scientific Research Group to guide COVID‐19 vaccines development. For instance, in 2023, the year after the adjusting of national strategy for prevent and control of SARS‐CoV‐2 in China, the vaccine research and development working group of the mechanism guided the variant COVID‐19 vaccines development as reserves for EUA.

The well‐established laws, procedures and guidance supported the acceleration of review and approval (Table 2). Major regulatory agencies have put in place a comprehensive set of laws and procedures to speed up the supply of vaccines in response to the pandemic. Most of these procedures have been maturely applied in the approval of seasonal and pandemic influenza vaccines, which can effectively guarantee the availability and annual strain updating within the limited manufacture time.^9^ In 2019, before the outbreak of the COVID‐19 pandemic, and in the context of China's drug regulatory system reform, the Vaccine Administration Law of the People's Republic of China was promulgated and implemented.^10^ The law clarifies the applicable circumstances for EUA and that the health department of the State Council will make recommendations and the National Medical Products Administration organizes the argumentation and agree. The Provisions for Drug Registration stipulates that accelerated registration pathways include breakthrough therapies, conditional approval, priority review and approval, special approval procedures, etc.^11^ Formulated forward‐looking technical guidelines guaranteed the vaccine development and rapid iterative research and review of variant strains.^12^

**TABLE 2:** Expedited programs for COVID‐19 vaccines in World Health Organization (WHO), National Medical Products Administration (NMPA), Food and Drug Administration (FDA), and European Medicines Agency (EMA).

Program/agency	Type	Laws	Applicable condition	Application period	Features	Post‐marketing requirement	Cancellation	Validity/conversion	References
WHO	Emergency use listing procedure	‒	‐PHEIC ‐In the interest of public health ‐Serious or life‐threatening disease	Clinical stage	‐Rolling review ‐Pre‐submission meeting ‐Development should continue for MA/PQ	‐Establish post‐EUL monitoring mechanism	Quality/safety/efficacy not be satisfied	‐Validity 12 months ‐Reassess within 12 months	^13^
NMPA	Conditional approval procedure	‐Drug Administration Law ‐Vaccine Administration Law ‐Provisions for Drug Registration	‐Drugs treat serious diseases without effective therapeutic means ‐Drugs urgently needed in public health ‐Vaccines urgently needed for public health emergencies or urgently needed as identified by the National Health Commission ‐Benefits outweigh risks	Clinical stage	‐Pre‐submission interaction ‐Priority review and approval can be applied ‐130 days or 70 days for drugs for rare disease in urgent needs marketed overseas	‐MAH shall complete the new or ongoing drug clinical trial within the specified time limit in accordance with the specific conditions	‐Failing to complete the study and submit the supplementary application as required within the time limit	‐Fulfill the obligation ‐Submit supplementary application for full marketing approval	^14^, ^15^, ^16^, ^17^
	Special review and approval procedure	‐Provisions for Drug Registration	Therapeutic and prophylactic drugs corresponding to public health emergency	IND, clinical stage, NDA	‐Unified command ‐Early intervention ‐Rapid, efficient and scientific review and approval ‐Accelerate and concurrently carry out drug registration acceptance, review, inspection and testing ‐24 h working ‐Report the inspection result within 5 days ‐Report testing result within 2 days ‐Complete first review cycle within 15 days	‒	‒	‒	^14^, ^15^, ^16^, ^17^
	Emergency use authorization	‐Vaccine Administration Law	‐Serious public health emergency ‐Other serious threat to public health	Clinical stage	‐National Health Commission propose recommendation ‐NMPA organize the evaluation	‐Establish post‐EUA monitoring mechanism ‐Continue the development	‒	‐Within certain scope and period	^14^, ^15^, ^16^, ^17^
FDA	Priority view	Prescription Drug User Fee Act of 1992	‐Drug treats a serious condition ‐Improved drug in safety or effectiveness ‐Labeling change on pediatric study ‐ Drug qualified infectious disease	BLA, NDA, or efficacy supplement	‐Shorter clock for review of marketing application ‐6 months	‒	‒	‒	^18^, ^19^
	Emergency use authorization	FD&C Act	‐Serious or life‐threatening disease or condition ‐Evidence of risk‐benefit analysis ‐No alternatives	Clinical stage	‐Early interaction ‐Hours to days	‐Continue on‐going clinical trial	‐Once public health emergency over	Application	^18^, ^19^
EMA	Conditional marketing authorization	Reg. (EC) No. 726/2004 Reg. (EC) No. 507/2006	‐Serious or life‐threatening disease or condition ‐Evidence of risk‐benefit analysis ‐No alternatives	Clinical stage	‐Early dialogue ‐Rolling review ‐150 days	‐Post‐authorization safety study and post‐authorization efficacy study required to be conducted ‐Renewal annually	‒	‐Validity 1 years ‐Fulfill the obligation ‐Renewal or new‐submission to grant marketing authorization	^20^, ^21^, ^22^

Abbreviations: BLA, Biologics License Applications; EUL, Emergency Use Listing; IND, Investigational New Drug; MAH, Marketing Authorization Holder; NDA, New Drug Application; PHEIC, Public Health Emergency of International Concern.

The timely switching of technology platforms during the vaccine development process, as well as the self‐innovation of developers and the iteration of the technology platform itself, have promoted the improvement of COVID‐19 vaccines. For instance, as of adjuvanted recombinant protein vaccines developed based on the Sf9 cells expression system in China, the manufacturer developed both covalent dimeric protein adjuvanted with aluminum and self‐assemble trimeric protein adjuvanted with oil‐in‐water emulsion. Unlike the simply strain updating, the self‐reform or updating of technical platform might provide a promising ability to develop a better iterative COVID‐19 vaccines in a more time‐consuming and costly way.

## 3 STRATEGIES FOR ACCELERATING VACCINE DEVELOPMENT

Accelerating vaccine development should be an efficient pathway to realize the vaccine availability responding to public health events. Pathways to save time encompass utilization of innovative vaccine technologies and methods, establishment of the Correlate of Protection, surrogate endpoint, industrial guidance, and the application of regulatory platform concept. Specially enhancing collaboration among stakeholders, variant monitoring, and strains recommendation.

### 3.1 Utilization of innovative vaccine technologies

The development of vaccines remains a complex, time‐consuming, and costly process. The use innovative technologies and methods could provide a more efficient way. Current innovative technologies and methodologies for the vaccines coverage research and development, testing, and manufacturing processes.^23^, ^24^

For vaccine research and development, omics, reverse vaccinology, next generation platforms, and vaccination routes could be selected. For example, combining analyses of DNA, RNA, and proteins, using computer‐based pathogen gene analysis to discover potential antigens, utilizing delivery vehicles for antigen genes such as liposomes, and administering vaccines via dermal (skin) and mucosal (oral, nasal) routes. For vaccines testing, organ chips, artificial intelligence and machine learning, electronic health records, common control group, standardized assays, virtual clinical trials, and wearable devices could be selected. For vaccine manufacturing, single‐use systems, modular bioprocessing systems, cell‐free synthesis, process optimization, and continuous manufacturing systems could be selected.

With the advancement of science and technology progress in vaccine research and development, researchers and manufacturers should combine the innovative technologies for vaccine development, testing, and manufacturing to promote the rational, accurate, and efficient development of COVID‐19 vaccine to maximize vaccine efficacy while ensuring quality and safety in the public health event. Challenges encompass inherent technological limitations and maturity, high cost, lack of standards and highly trained personnels and limited stakeholder collaboration, etc., affect the adoption of innovative technologies and methodologies.

### 3.2 Establishing Correlate of Protection and surrogate endpoint

Immunological trials could save time comparing with efficacy clinical trials. A well‐established Correlate of Protection could serve as a surrogate endpoint for the vaccine efficacy. Many viral vaccines use vaccines‐induced antibody levels as surrogate endpoints, such as rabies vaccine, influenza virus vaccine, and hepatitis B vaccine.^25^, ^26^, ^27^

Neutralizing antibody and cellular immune responses of COVID‐19 vaccines induced varied from different platforms. Both the World Health Organization (WHO) and the major national regulatory agencies including EMA, NMPA, FDA, and MHRA indicated to investigate the relationships between neutralizing antibody levels and effectiveness in relevant guidance for COVID‐19 vaccines. Studies confirmed a correlation between the level of induced neutralizing antibodies responses and the protection level to SARS‐CoV‐2.^28^, ^29^ T‐cell response is vital to induce high‐affinity antibodies, immune memory, and associated with protection.^30^, ^31^, ^32^ The collaborative calibration of the First WHO International Standard and Reference Panel and the first Chinese national standards for SARS‐CoV‐2 antibody indicated the presence of differences among different laboratories and methods.^33^, ^34^

Nowadays, immune bridging trials were conducted to support the variant COVID‐19 vaccines authorizations for special platforms. Immune correlate of protection and standardized serological test methods should be continuously studied to assess and establish a surrogate endpoint.

### 3.3 Streamlining studies without compromising safety and efficacy

Guidelines for the variant vaccines are established. In 2021, guidance/reflection paper were issued by regulatory authorities to lay out scientific and regulatory considerations about the variant COVID‐19 vaccines modified from the authorized/approved prototype COVID‐19 vaccines.^18^, ^35^, ^36^ Established guidelines for variant vaccines were sent to relevant entities and manufacturers via a point‐to‐point way in China. For vaccines that meet certain prerequisites, some preclinical studies can be exempted, and bridging clinical trial research data can be provided to speed up the authorization or approval of variant vaccines. For modified vaccines, the relevant guidance stated that prioritizing only changing the antigen and introducing as few other changes as possible, with the use of platform data and priori knowledge to simplify studies and accelerate the development, authorization, or approval of variant vaccines.

Platforms concept is applied. In early phase of the pandemic, the approval of adenovirus vector prototype COVID‐19 vaccine derived from the approved Ebola vaccine platform in China, applied relevant concepts of emergency and platform in the WHO's Ebola vaccine guidelines. Platform concept was also indicated in the established variant COVID‐19 vaccine guidelines. FDA's rapidly authorization of variant vaccines was technically based on the clinical effectiveness and immunogenicity data of previous COVID‐19 vaccines from the same manufacturer and the platform using the same or very similar process, as well as related safety data. EMA used a platform approach to approve modified variant vaccines, with the only requirement of manufacturing/quality and non‐clinical data, and plans for post‐authorization monitoring of effectiveness and immunogenicity/safety. The MHRA explicitly considers updating variant vaccines based on the regulatory principles for seasonal influenza updates and based on sufficient non‐clinical or clinical data.

### 3.4 Collaboration among stakeholders

Establish a collaborative mechanism. For example, the joint prevention and control mechanism established in China, which coordinates various national government agencies and related enterprises, such as the Ministry of Industry and Information Technology, Center for Disease Control and Prevention, National Medical Products Administration, manufacturers, raw material suppliers, etc.

Optimize regulatory measures to bridge the development and approval. For example, the research‐review‐linked review method through the lifecycle of prototype COVID‐19 vaccines and on‐site office methods to solve problems adopted during the emergency.

Strengthen basic research data collection and sharing. For instance, in April 2023, a publication in China reflected vaccination with a vaccine containing XBB.1.5 can induce a strong and extensive immune response against XBB.1.5 and other common progeny variants of Omicron, and that the neutralizing antibodies titer is related to the number of RBD mutations negatively related. The study also predicted that vaccines containing components of XBB.1.5 would be the most promising vaccine candidates against common Omicron variants.^37^ This publication provided a good looking‐forward reference for the XBB variant vaccines’ development in China.

### 3.5 Enhancing variants monitoring and establishing strain recommendation mechanism

Applied official mature influenza surveillance systems to the SARS‐CoV‐2 to strengthen strain monitoring and establish a unified virus strain recommendation mechanism. The World Health Organization (WHO) has successfully established a robust SARS‐CoV‐2 strain surveillance and estimation system. Both the FDA, EMA, and WHO have been actively involved in recommending variant vaccine strains twice a year. Partly due to the background of different epidemic prevention and control strategies, vaccinations and infections, the variant prevalence reflected a time disparity in different regions. The recommended time for virus strains should consider about the cross‐protective effect of existing vaccines, the circulating and mutation trends of virus strains, population immune background in different regions, and the time required for updating different technology platforms. Developers need to continuously track the circulating variant strains and make necessary preliminary development work reserves, such as virus seeds.

## 4 REGULATORY CONSIDERATIONS IN THE POST‐PANDEMIC ERA

Different from the early emergency phase, data accumulated for COVID‐19 vaccine platforms. Platform technology assessing and application, real‐world data leveraging, modification regulatory pathways based on accelerating procedures and methods combined with influenza‐based supervision might be considered.

### 4.1 Detailed assessing and using of platform technology

Different manufacturer's platforms conducted different strain change cycles with various degrees of data accumulations. Due to the difference such as the numbers of vaccines approved or in development and the validation of clinical efficacy, the maturity and robustness of platforms vary from each other.

Next step, it might be necessary to make definition, grade and prioritize the platforms according to the maturity. The technical guideline for variant vaccines should be updated and established detailed principles for different technical routes based on the platform datasets. It will be a promising pathway to simplify or reduce application dossiers, process or method validation, clinical trial and GMP inspection, etc. More flexible and simplified requirements for development and approval of updated strain vaccines will be handled case‐by‐case efficiently, pursuant to relevant laws, procedures, and guidelines.

### 4.2 Leveraging real‐world data

A large amount of real‐world data are accumulated with the approved and emergency use of COVID‐19 vaccines developed with different platforms, which provides the possibility to make a comprehensive analysis and comparison. An effective application of the data can help streamline nonclinical and clinical studies or optimize clinical trial plans, especially for the iteration of variant vaccines. It seems that real‐world data were successfully integrated into lifecycle management of COVID‐19 vaccines, including boosters, safety considerations, and iteration vaccines authorizations.

For instance, FDA optimized the dosing regimens and enhance the target populations, granted the EUA of variant vaccines with post‐marketing efficacy and safety monitoring data. FDA authorized mRNA vaccines to expand the eligible population for the homologous and heterologous booster doses and expand the use as a single booster dose.^38^ The real‐world evidence of safety signal for TTS of the Janssen adenoviral‐vectored COVID‐19 vaccine led to a recommendation for using mRNA vaccines in the United States. FDA authorized the EUA of variant vaccines via observed efficacy data and the post‐marketing safety monitoring data of the last generation vaccines.^39^, ^40^

Although the real‐world evidence remains a rich source of safety and efficacy information, it still faces many challenges in the future. The generation of real‐world evidence necessitates a comprehensive approach encompassing the development of robust data infrastructure, active public engagement, harmonization of data, assurance of quality, and proactive planning for various scenarios. The role of phase III trials, immune bridging clinical trials and real‐world effectiveness in different conditions or scenario deserve further discussion.^41^

### 4.3 Potential modifications to regulatory pathways

Gradually apply the concept of influenza‐based supervision to the development and approval of COVID‐19 vaccines. With reference to the influenza vaccine regulatory model, brief the marketing application process. For instance, EMA has authorized the COVID‐19 vaccine with a conditional and standard marketing approval process, while variant strain updates are approved as adaptive authorization, and the strain update submit is handled as type II variation. FDA approved the mRNA vaccines with composition of XBB.1.5 through a supplement for 12 years of age and older.

## 5 CONCLUSION

Though different prevent and control strategies were adopted during the pandemic of SARS‐CoV‐2 among different countries or regions in the past years, during years of development and continuous updating, up to 2023, the availability of variant vaccines have been globally harmonized either the technical routes or the composition of variant. On April 26, 2024, WHO recommended COVID‐19 vaccines to use a monovalent JN.1 lineage antigen, or other formulations against currently circulating variants, particularly JN.1 descendent lineages.^42^ Additionally, in the vaccine prequalification priority list released by the WHO for 2024−2026, the COVID‐19 vaccine is listed as a potential vaccine candidate.^43^

Laws, procedures, guidelines, and special unified and coordinated working mechanism enable efficient regulatory process to approve or authorization and guarantee the availability of COVID‐19 vaccines. It is important to make further improvement of policies, laws and procedures, for instance, improve the detailed process of EUA.

Accelerating procedures and methods, innovative vaccine technologies might be applied in the development and approval of COVID‐19 vaccines continuously to promote the efficiency. Enhancing the studies to establish Correlate of Protection and surrogate endpoint, detailed degrading and evaluation of established platforms, utilization of platform technology and real‐world data will create a more flexible, simplified and efficient regulatory approach to streamline studies and optimize regulatory procedures without compromising safety and efficacy.

Considering the uncertain continuous evolution and epidemical rules of SARS‐CoV‐2, we should explore to establish the strain updating regulatory process for COVID‐19 vaccines and a unified strain recommendation working mechanism, and solid and improve the coordinated working mechanism. For instance, strengthening the variant surveillance and human serology investigation and keeping deep cooperation with stakeholders including health agencies, regulatory agencies, manufacturers, and academia to develop variant COVID‐19 vaccines, both modified vaccines and innovative vaccine. During the SARS‐CoV‐2 pandemic, WHO implemented vaccine solidarity clinical trials for the rapid development of global COVID‐19 vaccines. Multi‐vaccines, international multi‐centers, and shared placebo groups greatly save clinical trial resources. Solidarity trials could be effectively conducted organized at the national level, when the time‐limited vaccines are needed in response to a pandemic.

In general, the construction of development and regulatory ecological systems is the key to respond to the future unknown public health events effectively, including basic research, the seamless connection between virus monitoring, research and development, review and approval, manufacture, distribution, and post‐market surveillance.

## AUTHOR CONTRIBUTIONS

*Li Xu*: contributed to the conceptualization, data gathering, drafting, and revising of the manuscript. *Min Li and Wu He*: provided guidance for the manuscript. All authors have read and approved the final manuscript.

## CONFLICT OF INTEREST STATEMENT

The authors declare they have no conflicts of interest.

The authors received no specific funding for this work.

## ETHICS APPROVAL

Not applicable.

## Data Availability

Not applicable.
